# National Consumption of Antimicrobials in Tanzania: 2017–2019

**DOI:** 10.3389/fphar.2020.585553

**Published:** 2020-10-30

**Authors:** Romuald Mbwasi, Siana Mapunjo, Rachel Wittenauer, Richard Valimba, Kelvin Msovela, Brian J. Werth, Akida Msallah Khea, Emmanuel Alphonce Nkiligi, Edgar Lusaya, Andy Stergachis, Niranjan Konduri

**Affiliations:** ^1^ St. John’s University of Tanzania, Dodoma, Tanzania; ^2^ Ministry of Health, Community Development, Gender, Elderly, and Children, Dodoma, Tanzania; ^3^ Department of Global Health, School of Public Health, University of Washington, Seattle, WA, United States; ^4^ USAID Medicines, Technologies, and Pharmaceutical Services (MTaPS) Program, Management Sciences for Health, Dar es Salaam, Tanzania; ^5^ Dodoma Regional Referral Hospital, Dodoma, Tanzania; ^6^ Department of Pharmacy, School of Pharmacy, University of Washington, Seattle, WA, United States; ^7^ Tanzania Medicine & Medical Devices Authority, Dodoma, Tanzania; ^8^ USAID Medicines, Technologies, and Pharmaceutical Services (MTaPS) Program, Management Sciences for Health, Arlington, VA, United States

**Keywords:** antibiotic consumption, antimicrobial consumption, Tanzania, surveillance, defined daily doses, private sector, Sub-Saharan Africa

## Abstract

**Objective:** Surveillance of antimicrobial consumption is essential to the national action plan for antimicrobial resistance (AMR) as stipulated in the Global Action Plan on AMR and the Tanzanian National Action Plan on AMR. Given the paucity of antimicrobial consumption data in sub-Saharan Africa region, the objective of this study was to measure antimicrobial consumption in Tanzania.

**Methods:** From 2017 to 2019, data on all antimicrobials imported into Tanzania were obtained from the Tanzania Medicines and Medical Devices Authority Data, augmented with purchasing data from the Medical Stores Department and data from local manufacturers. Data were collected and analyzed in accordance with the World Health Organization Anatomical Therapeutic Chemical and defined daily doses (DDD) methodology.

**Results:** The average DDD per 1,000 inhabitants per day (DDD/1,000/D) for all antimicrobials was 80.8 ± 39.35. The DDD/1,000/D declined from 136.41 in 2017 to 54.98 in 2018 and 51.02 in 2019. Doxycycline, amoxicillin, and trimethoprim-sulfamethoxazole were the most frequently consumed antibiotics during these years, accounting for 20.01, 16.75, and 12.42 DDD/1,000/D, respectively. The majority of antimicrobial consumption in Tanzania occurred in the private sector, with the proportion of private-sector antibiotic consumption increasing annually from 2017 to 2019. Based on AWaRe classification >90% of antimicrobial consumption was Access class medications, with Watch and Reserve class medications accounting for <10% and <1%, respectively.

**Conclusion:** The private sector use of antimicrobials is significantly increasing and should be carefully monitored in accordance with national policies. Future work is necessary to increase reporting of antimicrobial consumption patterns in sub-Saharan Africa.

## Introduction

The Global Health Security Agenda (GHSA) is a global partnership of countries and organizations that aim to make the world safe from infectious disease threats by raising countries’ capacity to strengthen global health security. The GHSA uses the World Health Organization’s (WHO) Joint External Evaluation tool to map and measure progress in country capacity in various technical areas. The GHSA supports antimicrobial resistance (AMR) containment through its AMR Action Package, one of the 19 technical areas addressed by the agenda (Global Health Security Agenda). The United Republic of Tanzania was rated as having “no capacity” (score 1) for antimicrobial stewardship in 2016 (World Health Organization, 2017). In response, Tanzania’s National Action Plan on AMR has laid out a multipronged plan for antimicrobial stewardship that includes establishing antimicrobial consumption surveillance at all levels of the health system (The United Republic of Tanzania, 2017b).

Data on antimicrobial consumption help provide a benchmark to enable policy makers to better understand priorities for antimicrobial stewardship (Van Boeckel et al., 2014; Versporten et al., 2014) and track the effectiveness of interventions, including changes in policies and guidelines (Kim et al., 2018; Wushouer et al., 2020). The WHO Global Action Plan on AMR seeks, among other goals, to reduce global human consumption of antibiotics while ensuring access to and optimizing the use of antimicrobials (World Health Organization, 2015). In 2016, Tanzania was one of four sub-Saharan African countries that participated in WHO’s first global program on surveillance of antimicrobial consumption among 65 countries (World Health Organization, 2018). Since this analysis of the 2016 data, there has been no further surveillance of antimicrobial consumption reported in Tanzania at the national level. The WHO Benchmarks for International Health Regulations recommends that member states establish protocols and databases for monitoring antimicrobial use and consumption for a country to reach capacity levels 2 and 3 (World Health Organization, 2019b). The objective of this study was to determine the national consumption of antimicrobials in Tanzania. We report a 3-year trend analysis of antimicrobial consumption by the public and private sectors.

## Methods

### Study Design

The data were collected and analyzed in accordance with the WHO Anatomical Therapeutic Chemical (ATC) and defined daily doses (DDD) methodology (World Health Organization, 2016). The ATC/DDD system is maintained by the WHO Collaborating Centre for Drug Statistics Methodology (2019). The Antimicrobial Consumption (AMC) tool was used to assign ATC codes and calculate DDD (WHO Collaborating Centre for Drug Statistics Methodology, 2020).

### Sampling

Data on all antimicrobials imported into Tanzania were obtained from the Tanzania Medicines and Medical Devices Authority (TMDA). The TMDA regulates and approves antimicrobials that are imported in the country by the Medical Stores Department (MSD)—the main agency responsible for the supply of health commodities to the public sector; private importers; and individual organizations and public institutions (e.g., hospitals, research institutions, donors) that import directly. The TMDA issues all import permits for any medicine imported into the country. As soon as they are paid for, all approved import purchase orders are entered into the TMDA import permit data system. The products are entered based on various categories (e.g., medicines, medical supplies) with details such as product strength, form, pack size, and total quantity. Additional data were obtained from Tanzania-based manufacturers that produce antimicrobials for local consumption (i.e., quantities only intended for sale to the local market).

The TMDA has a data system known as Information Management System where data were readily available from the system and accessed after acquiring authorization and being granted the login credentials.

Data on antimicrobial consumption were obtained for January 2017–November 2019. Substances included were all J01 (antibacterials for systemic use), J04A (antimycobacterials for treatment of tuberculosis), and P01AB (nitroimidazole derivatives). Though A07AA (antibiotics for intestinal tract) products are considered a core class of antimicrobials in the WHO protocol, they are not included in our results because they were not consumed in Tanzania during the period of analysis. Data from the TMDA and MSD were received as MS Excel files.

### Measures

The measures of interest for this analysis consisted of the consumption of antimicrobials across classes of interest by year; substance; route of administration; public vs. private sector; and Access, Watch and Reserve (AWaRe) categorization. The analysis included reporting on the most frequently used antimicrobials by pharmacological class (ATC03). The substances are also listed by AWaRe categorization of antimicrobials using both the WHO classification and the context-specific Tanzania Ministry of Health, Community Development, Gender, Elderly, and Children (MoHCDGEC) AWaRe classification (The United Republic of Tanzania , 2017a; World Health Organization, 2019a). The AWaRe classification was first applied in 2017 in Tanzania’s standard treatment guidelines based on the level of health system. The access group of antibiotics is to be used in dispensaries and health centers. The watch group of antibiotics is to be used in district and regional hospitals. The reserve group of antibiotics is to be used only at the tertiary level (i.e., national, zonal, referral, and specialized hospitals).

### Data Collection

**FIGURE 1:**
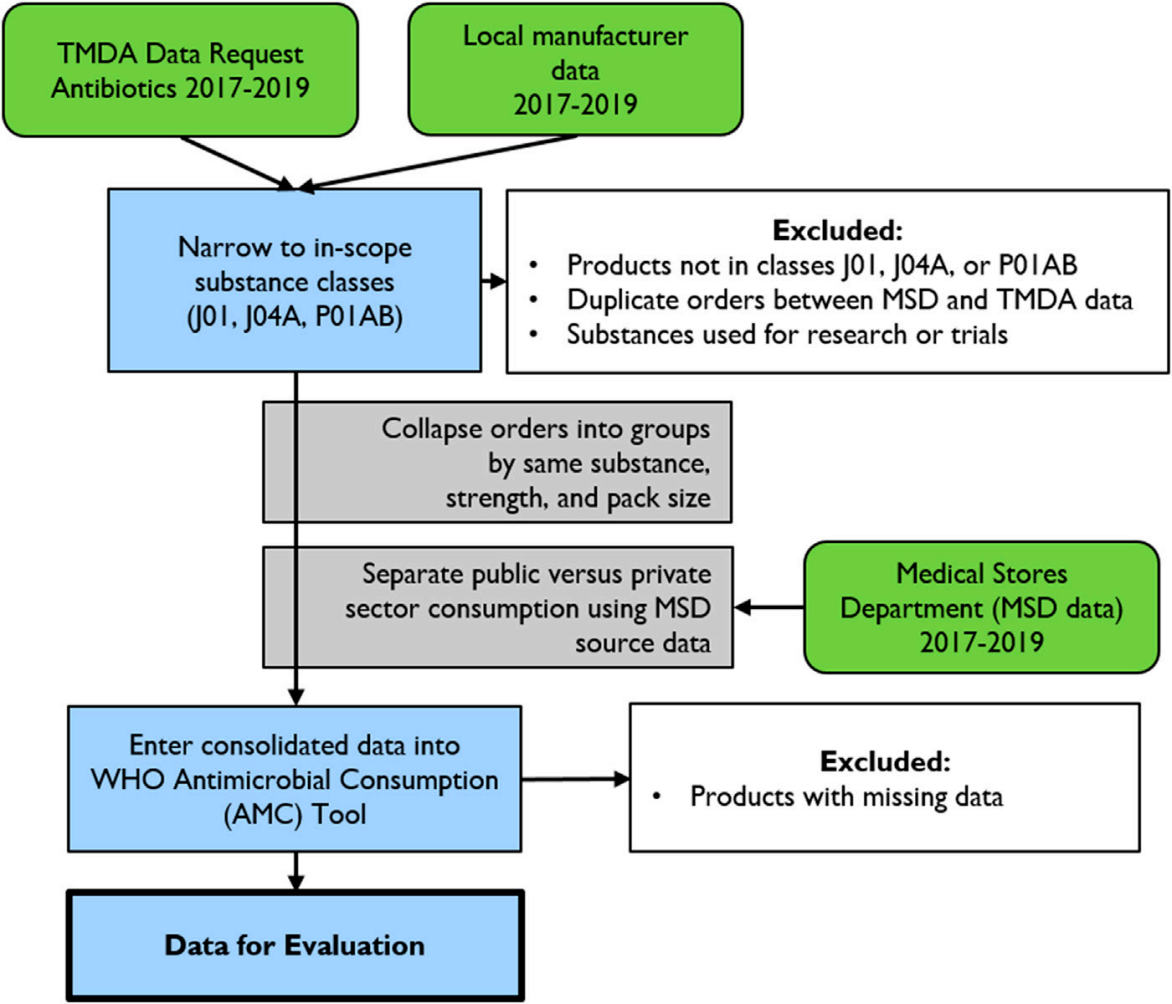
Data collection and cleaning methodology for the Tanzania national antibiotic consumption analysis.

A 2-day training workshop on national-level antimicrobial consumption analysis was conducted in December 2019 for 14 people from the MoHCDGEC (from the TMDA, MSD, and Pharmacy Services Unit). The training was performed by the authors (RM, SM, NK, BW, AS), and the content was based on WHO’s protocol for conducting the national-level antimicrobial consumption analysis using the WHO ATC-DDD methodology (World Health Organization, 2016). Data were collected between December 2019 and January 2020 by selected staff from the MoHCDGEC and supervised by RM, RV, and SM. The data collection team extracted all antimicrobials for in-scope substances from the TMDA database. Data on locally produced antimicrobials were collected at the manufacturer level, which provided total annual production of each antimicrobial and the quantities sold in the local market (Figure 1).

### Extraction and Categorization of National Consumption Data

Using the TMDA medicines import file, we identified all importers for 2017, 2018, and 2019 and created separate files for each year. We then extracted all relevant imported antimicrobials. The annual total for each product was calculated for each importer. The TMDA controls all imports of medicines in the country, and each importer, including the MSD and other public institutions, must get an import permit from the TMDA before importing any pharmaceutical product into the country. The sub-totals of individual products imported by all importers (public and private) were added to provide total national imported quantities for each product. Data were then collapsed by product type for each product with the same strength and pack size and grouped as tablets/capsules, parenterals, and suspensions/syrups. The total national imported quantities for each product were added to the total quantities of similar products locally produced by the four local antimicrobial manufacturers.

After combining individual product orders of the same substance, strength, and pack size from both the TMDA (all importers) and local manufacturers’ datasets, the data were entered into the WHO AMC tool (WHO Collaborating Centre for Drug Statistics Methodology, 2020). The AMC tool generated a second dataset with the product common name, unit pack size, strength in grams, ATC code, dosage form, and quantity (volume). The AMC tool also translated the consumption into DDD format and DDD per 1,000 inhabitants per day for analysis. The latter was calculated using population estimates for 2017–2019 (Worldometers Population Database).

### Categorization of the National Data Into Public and Private Sector

We observed that 1) among private importers, some sold part of their imported consignments to the MSD while others did not; and 2) the MSD also purchases antimicrobials from local manufacturers. Therefore, we identified each local importer from the TMDA file and all local importers that sold individual products to the MSD from the MSD file and subtracted the total quantities for products supplied to the MSD from the annual total imported quantities to get the quantities that remained in the private sector. Similarly, the MSD file contained data on quantities of antimicrobials bought from local manufacturers. We subtracted these quantities from annual total quantities of individual antimicrobials produced by each manufacturer to get the quantities that went to the private sector. After this basic process, t the public and private sector datasets were aggregated per each product and per type of formulation as they were for the national data and entered into the AMC tool. The final dataset was used for analysis of national antibiotic consumption in Tanzania between 2017 and 2019. Figure 1 summarizes the data collection and cleaning methodology.

### Data Analysis

We present totals and summary statistics for DDD per 1,000 inhabitants per day (DDD/1,000/D). Since data were collected in December 2019, we multiplied the 2019 data by 12/11 (1.09) to estimate consumption for the year. Data were analyzed using R Studio and Microsoft Excel.

## Results

### Overall National Consumption and by Year

The average total consumption of all antimicrobials in Tanzania was 1,508,664,371 DDDs per year from 2017 to 2019. The average DDD per 1,000 inhabitants per day (DDD/1,000/D) for all antimicrobials was 80.8 ± 39.35. The DDD/1,000/D declined over time, from 136.41 in 2017 to 54.98 in 2018 and 51.02 in 2019. The total DDD/1,000/D for each of the 3 years is summarized in Table 1 according to ATC01, sector of consumption, and AWaRe category.

**TABLE 1:** Antimicrobial consumption in DDD per 1,000 inhabitants per day by ATC01, including route of administration, sector of consumption, and AWaRe category.

	DDD/1000/day (%)			Mean ± SD
	2017	2018	2019	
Total	136.41 (100)	54.98 (100)	51.02 (100)	80.80 ± 39.35
J01 all	131.86 (96.7)	43.95 (79.9)	42.87 (84)	72.89 ± 41.70
J01 oral	127.84 (93.7)	43.28 (78.7)	41.7 (81.7)	70.94 ± 40.24
J01 parenteral	4.02 (2.9)	0.67 (1.2)	1.18 (2.3)	1.96 ± 1.47
J04 all (oral)	0.66 (0.5)	4.4 (8)	1.66 (3.3)	2.24 ± 1.58
P01 all (oral)	3.89 (2.9)	6.63 (12.1)	6.49 (12.7)	5.67 ± 1.26
Public-sector consumption	56.17 (41.2)	23.55 (42.8)	5.75 (11.3)	28.49 ± 20.87
Private-sector consumption	80.25 (58.8)	31.43 (57.2)	45.26 (88.7)	52.31 ± 20.54
WHO AWaRe classification^a^				
Access	115.67 (84.8)	32.5 (59.1)	27.76 (54.4)	58.64 ± 40.37
Watch	15.6 (11.4)	14.06 (25.6)	17.89 (35.1)	15.85 ± 1.57
Reserve	0 (0)	0 (0)	0 (0)	0 ± 0
Tanzania AWaRe classification^a^				
Access	121.2 (88.8)	33.33 (60.6)	34.83 (68.3)	63.12 ± 41.07
Watch	10.07 (7.4)	13.23 (24.1)	10.82 (21.2)	11.37 ± 1.35
Reserve	<0.001 (0)	<0.001 (0)	<0.001 (0)	<0.001 ± 0

DDD per 1,000 inhabitants per day was calculated based on Tanzania’s national population each year—2017, 54.6 million; 2018, 56.3 million; 2019, 58 million (Worldometers Population Database). DDD, defined daily doses.

a AWaRe categories do not sum to 100% because some substances do not have an AWaRe categorization by either Tanzania or WHO.

**FIGURE 2:**
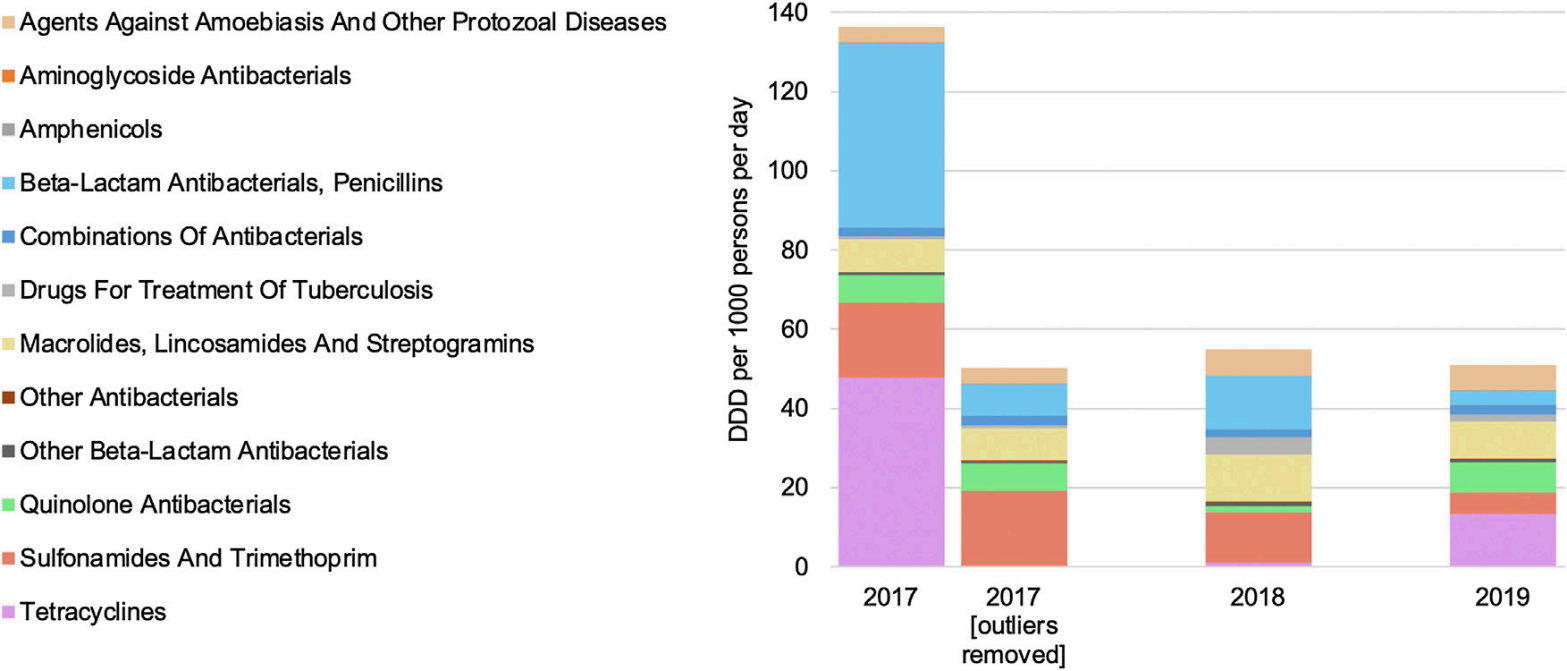
Antimicrobial consumption by year and Anatomical Therapeutic Chemical level.

Antibiotics for systemic use (J01) comprised the vast majority of antimicrobial consumption each year, accounting for 96.7%, 79.9%, and 84.0% of consumption in 2017, 2018, and 2019, respectively. Two outlier substances skew the 2017 data [tetracycline (J01A) and penicillin beta-lactam antibacterials (J01C)]. Except for the outlier values in 2017, national consumption of antimicrobials remained relatively constant over time (Figure 2).

In 2017, the amounts of tetracycline (J01A) and penicillin beta-lactam antibacterials (J01C) consumed were 6.7-fold and 5.5-fold higher than in subsequent years, respectively. This increased consumption in 2017 was driven entirely by two large purchases of doxycycline and amoxicillin, which were 4.2-fold and 48.7-fold greater than the average consumption of these substances in 2018 and 2019, respectively.

### Consumption by Substance

The most highly consumed antimicrobials in the J01 category of substances to ATC5 level from 2017 to 2019 are listed in Table 2 .

**TABLE 2:** Antibiotic consumption as mean DDD per 1,000 inhabitants per day (DDD/1,000/D) from 2017 to 2019 by antibiotic class.

Rank	Substance	ATC code	Mean DDD/1,000/D ± SD
J01—antibacterials for systemic use			
1	Doxycycline	J01AA02	20.01 ± 24.529
2	Amoxicillin	J01CA04	16.759 ± 19.538
3	Trimethoprim-sulfamethoxazole	J01EE01	12.422 ± 6.851
4	Erythromycin	J01FA01	7.159 ± 2.027
5	Ciprofloxacin	J01MA02	4.616 ± 3.058
6	Azithromycin	J01FA10	2.711 ± 0.311
7	Norfloxacin and tinidazole	J01RA13	2.265 ± 0.31
8	Combinations of penicillins	J01CR50	1.33 ± 0.337
9	Procaine benzylpenicillin	J01CE09	0.953 ± 1.144
10	Phenoxymethylpenicillin	J01CE02	0.808 ± 0.816
11	Levofloxacin	J01MA12	0.707 ± 0.466
12	Tetracycline	J01AA07	0.655 ± 0.732
13	Amoxicillin and beta-lactamase inhibitor	J01CR02	0.452 ± 0.105
14	Benzylpenicillin	J01CE01	0.414 ± 0.646
15	Ceftriaxone	J01DD04	0.372 ± 0.049
16	Ampicillin	J01CA01	0.252 ± 0.345
17	Cefalexin	J01DB01	0.241 ± 0.164
18	Chloramphenicol	J01BA01	0.194 ± 0.044
19	Cefixime	J01DD08	0.101 ± 0.045
20	Ampicillin and beta-lactamase inhibitor	J01CR01	0.087 ± 0.123
J04—antimycobacterials			
1	Rifampicin and isoniazid	J04AM02	1.458 ± 1.657
2	Isoniazid	J04AC01	0.752 ± 0.297
3	Ethambutol	J04AK02	0.017 ± 0.021
4	Pyrazinamide	J04AK01	0.01 ± 0.017
5	Rifampicin	J04AB02	0 ± 0
6	Clofazimine	J04BA01	0 ± NA
P01AB—nitroimidazole antiprotozoal agents			
1	Metronidazole (parenteral)	P01AB01	5.248 ± 1.723
2	Tinidazole	P01AB02	0.387 ± 0.231
3	Secnidazole	P01AB07	0.024 ± 0.005
4	Ornidazole	P01AB03	0.007 ± 0.013

ATC, Anatomical Therapeutic Chemical; DDD, defined daily doses.

Doxycycline, amoxicillin, and trimethoprim-sulfamethoxazole were the most frequently consumed antibiotics over this time period, accounting for 20.01, 16.759, and 12.422 DDD per 1,000 inhabitants per day, respectively. Among the P01AB substances (nitroimidazole antiprotozoal agents), metronidazole was by far the highest volume consumed from 2017 to 2019. Antimycobacterial agents, or J04 substances, were consumed at much lower quantities than other antimicrobials. Isoniazid was the most common antimycobacterial agent and was consumed at least 17 times more frequently than the next highest-consumed antimycobacterial, pyrazinamide.

### Consumption in Public vs. Private Sectors

The percent of total antimicrobial consumption in the public vs. private sector in Tanzania from 2017 to 2019 is shown in Table 1. Overall, the 3-year average public-sector consumption accounted for approximately 35% of the total consumption and decreased from 41.2% in 2017 to 11.3% in 2019. Over the same time period, the 3-year average private-sector consumption accounted for approximately 65% of the total consumption and increased from 58.8% in 2017 to 88.7% in 2019.

**FIGURE 3:**
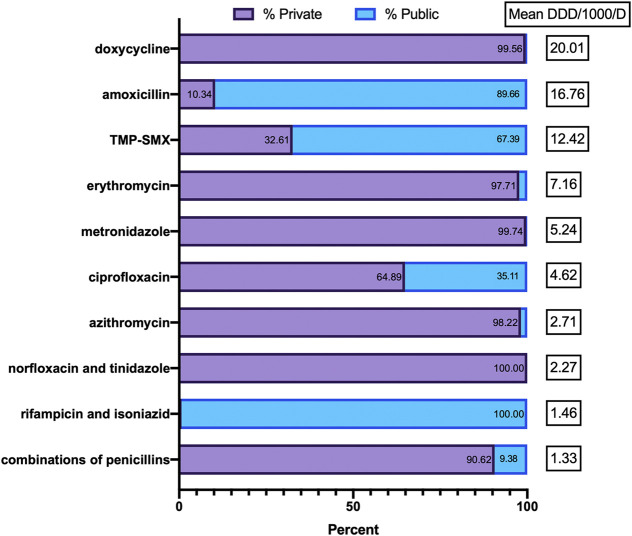
Proportion of public vs. private sector use of the top 10 antimicrobials.

The most common substances consumed by the public and private sectors are described in Figure 3. The pattern of antimicrobial consumption varied significantly between the private and public sectors. From 2017 to 2019, the private sector consumed more than 97% of all tetracyclines (doxycycline and tetracycline), macrolides (J01FA), and metronidazole, while the public sector consumed the vast majority of amoxicillin (89.7%). Differences in consumption between sectors were smaller among other antimicrobial classes, with other top 10 substances being used at similar rates. Over the 3 years studied, the private sector consumed 88.4% of the total DDD of parenteral antimicrobials and 64.0% of the total DDD of oral antimicrobials, while the public sector made up the remaining 11.6% and 36.0%, respectively.

### Consumption by AWaRe Classification

Table 1 describes antimicrobial consumption by WHO AWaRe classification as well as the Tanzania AWaRe classification, which is based on the WHO system but with some modifications to align with Tanzania’s national goals. Overall, the vast majority (>90%) of antimicrobial consumption was Access class medications, with Watch class medications accounting for less than 10% and Reserve class medications making up less than 1%. Erythromycin was the most commonly consumed Watch class medication (7.159 DDD/1,000/day), while clindamycin was the most commonly consumed Reserve class medication (<0.001 DDD/1,000/day).

### Consumption by Route of Administration

**FIGURE 4:**
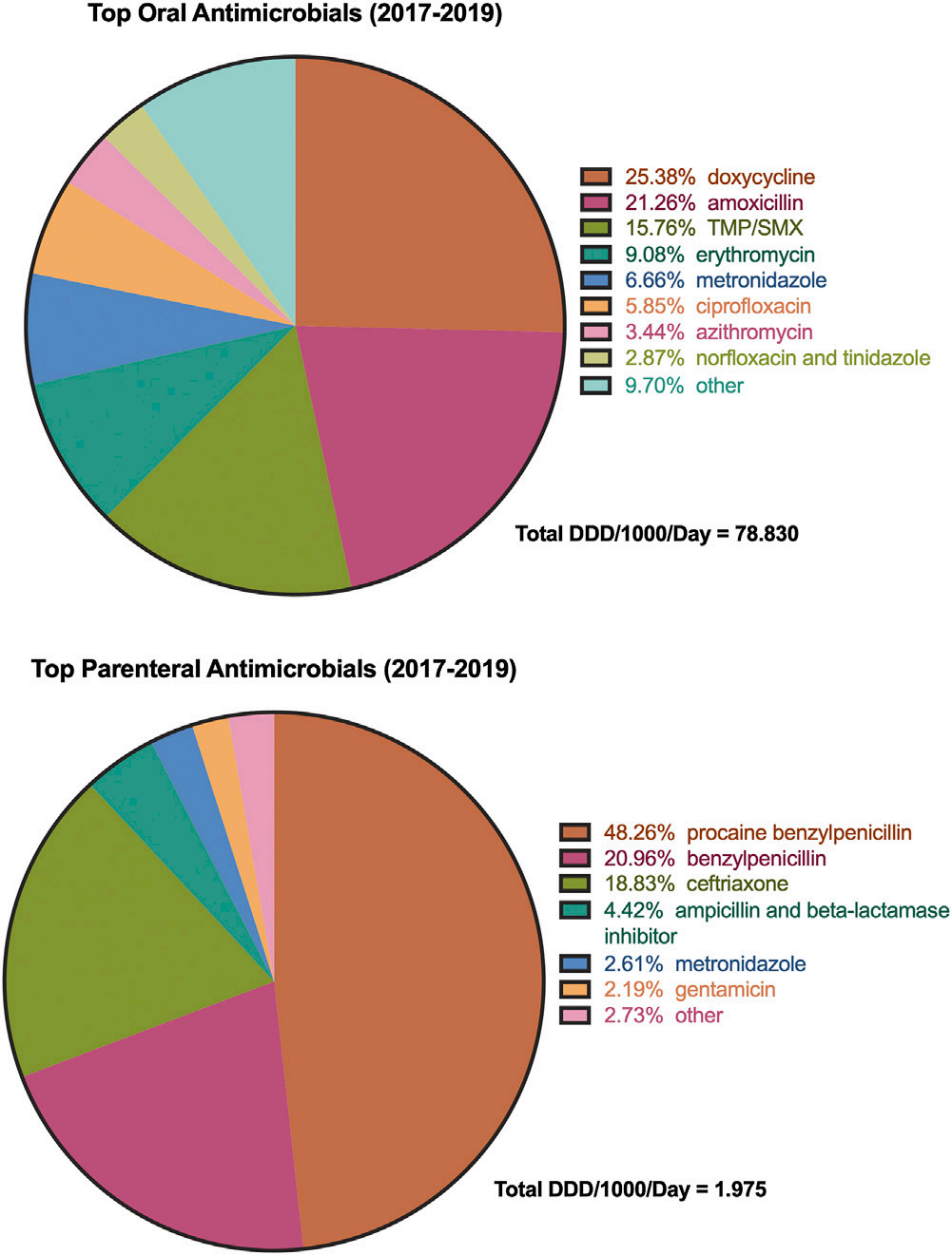
Most commonly consumed antimicrobials on average from 2017 to 2019 by route of administration. TMP-SMX, trimethoprim-sulfamethoxazole.

The most commonly consumed antimicrobials by route of administration are illustrated in Figure 4. Among the orally administered agents, amoxicillin and doxycycline comprised more than 50% of all substances. Nine agents accounted for approximately 95% of all oral antimicrobial consumption, while the other 31 agents made up the last 5%. More than 90% of all parenteral antimicrobial consumption was attributed to benzylpenicillin with and without procaine (75%) and to ceftriaxone (16%). Gentamicin and metronidazole each accounted for approximately 3%, while 27 other agents made up the final 3% of the total parenteral antimicrobial consumption.

## Discussion

To our knowledge, this is the first published study from the sub-Saharan Africa region that provides a comprehensive picture of national-level consumption of priority antimicrobials based on the WHO AMC methodology. A study in South Africa utilized private sector sales data and tender data from the public sector to analyze the compounded annual growth rate of various antimicrobial categories (Schellack et al., 2017). Ongoing measurement of national patterns in antimicrobial consumption is an essential part of monitoring the effectiveness of antimicrobial stewardship programs and national policies and guidelines aimed at reducing unnecessary antimicrobial use, thereby addressing contributing factors to antimicrobial resistance. It also allows for comparisons at the global or country level and raises awareness about the use of antimicrobials among health professionals, the general public, and policy makers. In this study, we report the national consumption of antimicrobials in Tanzania over the 3-year period of 2017–2019. With the exception of 2017, we found that national consumption of antimicrobials remained relatively constant over time. Tanzania’s antibiotic consumption for 2017 was considerably higher than what was observed for 2018 and 2019. Additionally, based on import records alone, Tanzania reported a much lower antibiotic consumption of 27.29 DDD per 1,000 inhabitants per day for 2016 compared to our findings for 2017–2019 (World Health Organization, 2018). When tracing back to the source data from the TMDA and MSD, there was no one single large order that stood out for 2017. Rather, the totals are aggregates of several large orders and do not appear to be a data entry error or otherwise explainable phenomenon. One possibility could be the significant increase of allocation for medicines in the 2017–2018 public health sector budget (Lee and Tarimo, 2018). High levels of reported resistance to doxycycline and trimethoprim-sulfamethoxazole and its identification as a target for antimicrobial resistance surveillance in humans in Tanzania suggest a possible reason for its annualized decreased use during the study period (Hall et al., 2020; The United Republic of Tanzania, 2018). Inappropriate use of amoxicillin and its use with clavulanate to combat generally high levels of resistance to penicillins may suggest a decreased use of amoxicillin in the study period (Mboya et al., 2018; Tadesse et al., 2017).

Overall, the findings also indicate that the majority of antimicrobial consumption in Tanzania occurs in the private sector and that the proportion of private-sector antibiotic consumption increased annually from 2017 to 2019. This phenomenon may be explained by progressively higher volumes of private-sector pharmaceutical imports over the years, with forecasted growth of 28% by 2021 compared to the previous 5 years (Wande et al., 2019). With the exception of requirements for all national health insurance fund-approved providers to adhere to Tanzania’s national standard treatment guidelines, other private-sector providers are not obliged, and therefore prescribing habits vary. However, the vast majority of antimicrobial consumption is in accordance with WHO’s target of at least 60% of and Tanzania’s Access component of the AWaRE classification (World Health Organization, 2019c).

The uniform methodology from WHO allows for comparisons of national trends across different countries. Reviewing recent antimicrobial consumption reports from the European Union, Japan, and China, we found that the overall consumption of antimicrobials in Tanzania (80 DDD/1,000/D) was greater than in the EU (18.4 DDD/1,000/D; 2018); Japan (14.1 DDD/1,000/D; 2004–2016); and China (9.1 DDD/1,000/D; 2017) (European Centre for Disease Prevention and Control, 2019; Tsutsui et al., 2018; Zhang et al., 2019). Factors that might explain the higher use of antimicrobials in Tanzania compared to other countries with recent reports may include a relatively higher burden of infectious diseases, limited diagnostic availability at the health facility level and point of care, widespread availability of antibiotics without prescription, and unexplained use of certain antibiotics in the animal health sector. We observed that the most common class of antimicrobial consumed in Tanzania was the tetracyclines (J01A), while the most common classes were beta-lactams (J01C) in the EU and China and macrolides (J01F) in Japan.

The study findings have several policy implications for national authorities in line with Tanzania’s national action plan on AMR. Health facilities must be directed to establish separate electronic data systems for all antimicrobials used. Major zonal and regional referral hospitals should be encouraged to triangulate data from local AMR sensitivity patterns with antimicrobial consumption and to compare the findings with standard treatment guidelines. All importers, wholesalers, distributors, and local manufacturers need to be directed to establish mandatory electronic systems on antimicrobials sold and distributed to enable easier tracking of antimicrobial consumption. Future research can then be easily done to track consumption in the entire supply chain down to the user level at health facilities.

A strength of the present study was the inclusion of records from local manufacturers as well as records from national medical stores. By including both imported and locally produced antimicrobials, these data present a comprehensive view of all antimicrobials consumed in Tanzania, which includes the public and private sectors.

This study had certain limitations. We did not include WHO’s optional ATC classes such as antifungals (J02), antivirals (J05), and antimalarials (P01B). Given limited resources, we focused on the study antimicrobials that was also of priority for the MoHCDGEC. Data used for this analysis were derived from import and purchasing records and may not reflect what is actually being consumed. For that information, data on the prescription, dispensing, and use of antimicrobials at the patient level are required. Another potential limitation is that some local manufacturers, distributors, and importers may export medicines that they produce, which would overstate national consumption estimates as this practice was not accounted for in our study. However, local manufacturers accounted for only 10% of antimicrobial consumption in our study. This methodology assumes that antibiotics are consumed in the same year they are produced, which may not be the case with these data given the lack of consistency in volume and type of product each year of analysis. Comparability of these data to other countries or settings may be limited without taking into account differences in age distributions between countries. The DDD methodology itself has certain limitations. For example, the methodology does not include data from purchases of antibiotics from informal markets. Further, data based on imports do not guarantee that all imported antimicrobials are used locally and not exported or bought by neighboring countries. Additionally, the DDD classification is not suitable for quantifying the consumption of antimicrobials in children, who often receive smaller weight-based doses compared to adults. In Tanzania, there appears to be wide variation in amount and substance of antimicrobials imported each year, making it difficult to draw conclusions and identify meaningful trends going forward with regard to consumption of certain substances.

## Conclusion

The steady increase in private-sector antimicrobial consumption suggests the need for close monitoring of antimicrobial use in private-sector settings in accordance with Tanzania’s national action plan on AMR. Assessment of antibiotic consumption helps provide a benchmark to enable policy makers to better understand priorities for antimicrobial stewardship and track the effects of interventions, including changes in policies and guidelines. Future work is necessary to better understand antimicrobial consumption trends in Sub-Saharan Africa.

## Data Availability Statement

Data are available upon reasonable request from the national AMR focal person in the Ministry of Health, Community Development, Gender, Elderly and Children.

## Author Contributions

RM, SM, RV, AS, BW, EL, and NK had substantial contributions to the conception and design of the work. SM, AS, and BW led the overall training of the research assistants. RM, SM, and RV supervised the data collection. RM, SM, and KM participated in the data collection. RM, KM, and RW played a critical role in the data management and data analysis. RW wrote the initial draft of the manuscript. RM, SM, RW, RV, BW, AMK, EAN, EL, AS, and NK critically reviewed and edited the draft manuscript. SM, RV, EL, and NK were responsible for coordinating all research activities and stakeholder engagement. All authors approved the final draft.

## Conflict of Interest

BW has received grant support from commercial sources including Shionogi Inc.

The remaining authors declare that the research was conducted in the absence of any commercial or financial relationships that could be construed as a potential conflict of interest.
